# Diagnostic pitfalls in constrictive pericarditis coexisting with right ventricular outflow tract obstruction and coronary artery anomaly

**DOI:** 10.1093/jscr/rjag132

**Published:** 2026-03-22

**Authors:** Mahmood Hosseinzadeh Maleki, Hoorak Poorzand, Ali Eshraghi, Navid Abbasiyan Fallahi

**Affiliations:** Department of Cardiac Surgery, Imam Reza Hospital, Mashhad University of Medical Sciences, Mashhad, Iran; Patient Safety Research Center, Clinical Research Institute, Mashhad University of Medical Sciences, Mashhad, Iran; Department of Cardiovascular Diseases, Faculty of Medicine, Mashhad University of Medical Sciences, Mashhad, Iran; Department of Cardiac Surgery, Razavi Hospital, Mashhad, Iran

**Keywords:** heart failure, pulmonary valve stenosis, left anterior descending artery, computed tomography angiography, case report

## Abstract

Constrictive pericarditis associated with an anomalous left anterior descending artery is a very rare and complex condition. The aim of this report is to highlight the diagnostic challenges and surgical management in this disease. A 40-year-old man with right heart failure, dyspnea, and ascites, underwent comprehensive cardiac evaluation and surgery. The pulmonary valve was dysplastic, causing severe right ventricular outflow tract (RVOT) obstruction. During resection, the left anterior descending artery was inadvertently transected and bypassed with a saphenous vein graft. RVOT reconstruction, pulmonary valve replacement, and tricuspid valve repair were performed. Postoperative follow-up showed satisfactory ventricular and valve function; however, structural changes and reduced right ventricular function persisted. Non-invasive coronary imaging is necessary when angiography is not feasible, and the limitations of transesophageal echocardiography should be considered. Chronic right ventricular changes may not be fully reversible, emphasizing the need for long-term follow-up.

## Introduction

Constrictive pericarditis is a rare and important disorder causing right heart failure, in which the pericardium becomes thickened, fibrotic, or calcified, leading to restriction of diastolic filling and the occurrence of dyspnea, ascites, and heart failure [1, 2]. On the other hand, right heart failure secondary to right ventricular outflow tract (RVOT) obstruction, pulmonary valve (PV) stenosis, or severe tricuspid regurgitation (TR) may also produce similar manifestations, and in patients with right ventricle (RV) pressure overload, differential diagnosis becomes challenging. In such conditions, accurate preoperative haemodynamic assessment and imaging are of fundamental importance [3, 4].

Furthermore, recognition of congenital coronary artery anomalies is of special importance due to changes in blood flow patterns and potential clinical consequences. Among these, recognition of left anterior descending (LAD) artery anomalies before surgery using computed tomography angiography (CTA) or other imaging methods is extremely important, because failure to detect them may increase the risk of injury to the artery during open heart surgery or RVOT related interventions [5].

In this case report, we present the diagnostic and surgical challenges in a rare case of constrictive pericarditis associated with severe RVOT obstruction, TR, and LAD anomaly, emphasizing the necessity of comprehensive preoperative evaluation.

## Case presentation

A 40-year-old male with a history of advanced heart failure and multiple hospitalizations presented to the emergency department with ascites, nausea, vomiting, severe weight loss, and obvious respiratory distress, without classic chest pain. On initial evaluation, he was classified as New York Heart Association (NYHA) functional class IV. The patient had no history of cardiac surgery or interventions, and no cardiac murmurs were auscultated on physical examination. Electrocardiogram demonstrated atrial fibrillation. Initial laboratory tests revealed elevated liver and renal enzymes.

Chest computed tomography showed cardiomegaly and pericardial effusion. Hepatic and biliary ultrasonography suggested secondary cirrhotic changes due to right heart failure, along with increased right atrial (RA) pressure.

**Figure 1:**
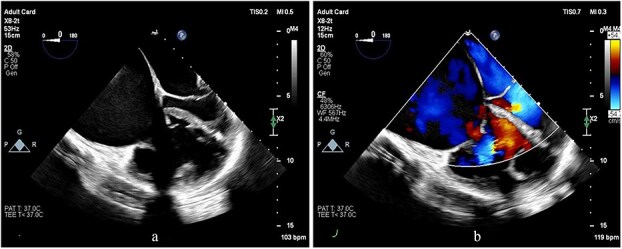
TEE images at the mid-esophageal level, four-chamber view, obtained with clockwise rotation focusing on the right heart. (a, b) The images demonstrate severe RV and RA enlargement, dilated tricuspid annulus, and functional TR. Note the leftward shifting of the interatrial septum toward the LA cavity, consistent with elevated RA pressure.

**Figure 2:**
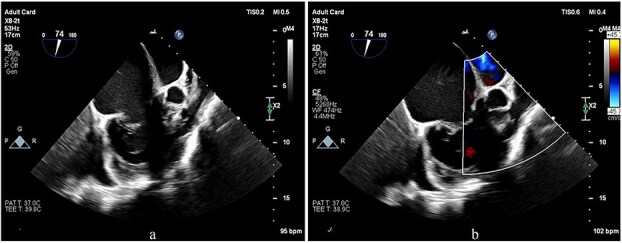
TEE images focusing on the RVOT and PV. (a, b) The images show doming of the PV, a prominent muscular bundle beneath the PV, and supravalvular narrowing, resulting in flow acceleration across all these levels.

**Figure 3:**
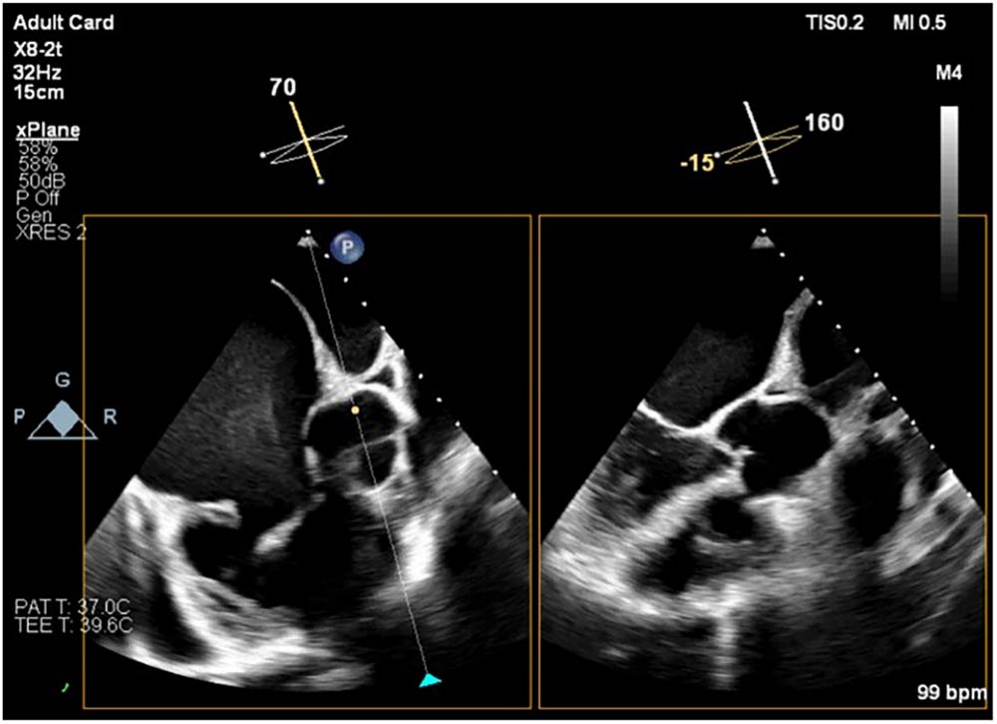
TEE image showing the short-axis view of the aortic valve and the corresponding long-axis view of the LV. There is no evidence of aortic overriding or septal defect. Note the spontaneous echo contrast smoke, representing slow blood flow, observed in the RA cavity.

Preoperative transesophageal echocardiography (TEE) demonstrated marked RV and RA enlargement, severe RV systolic dysfunction, thickened RV walls, severe TR due to annular dilation, and severe PV stenosis. No ventricular septal defect was observed preoperatively (Figs 1–3). The left ventricle (LV) appeared small and D-shaped, consistent with elevated RV pressures (Table 1).

**Table 1:** Key echocardiographic parameters before and after surgery

Parameters	Pre-operatively	Post operatively	Normal value	Interpretation
RVEDD (cm)	5.8	5	< 4.2 cm	Slight reduction
TAPSE (cm)	0.8	0.9	≥ 1.7 cm	Mild improvement
RV systolic function	Severely reduced	moderately reduced	Normal RV function	Partial recovery
RVOT VTI / PV gradients	PV PPG: 100 mmHg	PV PPG: 45 mmHg	< 25 mmHg	Significant relieved after PVR
SPAP (mmHg)	Not measurable / estimated	50	≤ 35 mmHg	Decreased pressure, mild PH persists
EF LV (%)	45	50	≥ 55%	LV function Improved

Abbreviations: RVEDD = Right Ventricular End-Diastolic Diameter; RV = Right Ventricle; TAPSE = Tricuspid Annular Plane Systolic Excursion; RVOT VTI = Right Ventricular Outflow Tract Velocity Time Integral; PV PPG = Pulmonary Valve Peak Pressure Gradient; PVR = Pulmonary Valve Replacement; SPAP = Systolic Pulmonary Artery Pressure; PH = pulmonary hypertension; EF = Ejection Fraction.

**Figure 4:**
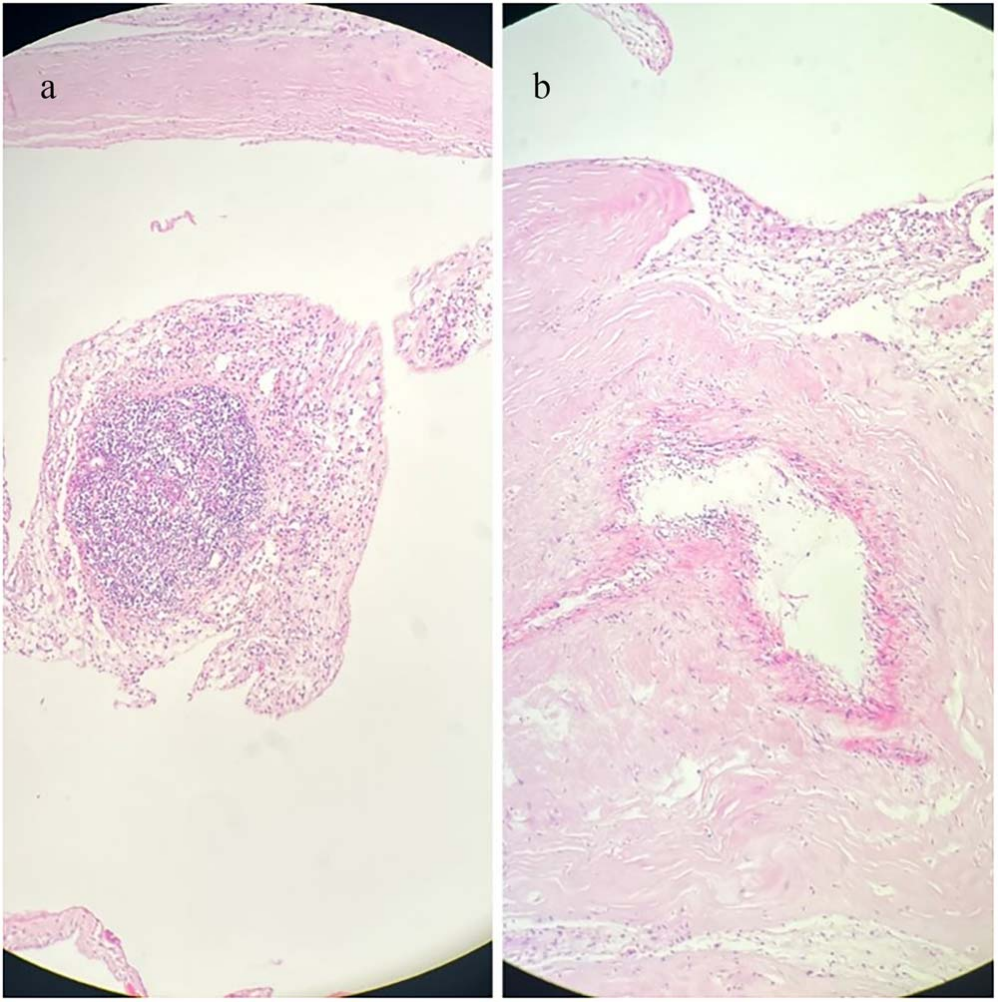
Histopathological sections of the pericardial biopsy stained with haematoxylin and eosin (H&E). (a) Dense granulation tissue with inflammatory cell infiltration. (b) Chronic fibrosing pericarditis characterized by thickened fibrous tissue. Scale bar not available.

**Figure 5:**
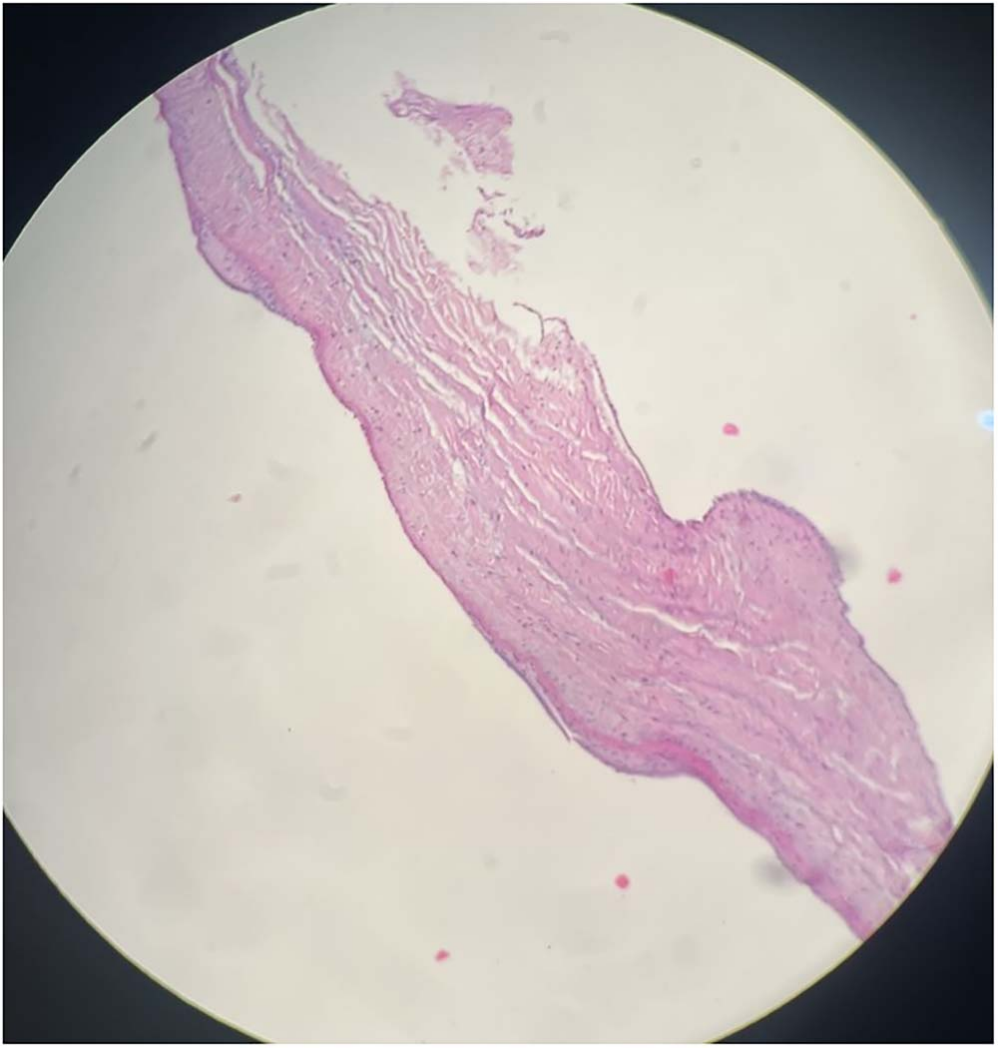
Histopathological section of PV biopsy stained with haematoxylin and eosin (H&E). The section shows degenerative fibrohyalinized valvulopathy of the PV. Scale bar not available.

Coronary angiography revealed right coronary artery and LAD without significant stenosis. Right heart catheterization confirmed significantly elevated RA and RV pressures; however, pulmonary artery pressure could not be accurately assessed due to severe PV stenosis. Following induction of general anaesthesia, a median sternotomy was performed. The pericardium was thickened, edematous, and adherent to the epicardium, necessitating extensive pericardiectomy. Biopsy of the pericardium and tissue beneath the PV revealed chronic fibrosis with granulation tissue formation and fibrotic/hyaline degeneration of the PV without evidence of tuberculosis (Figs 4 and 5).

**Figure 6:**
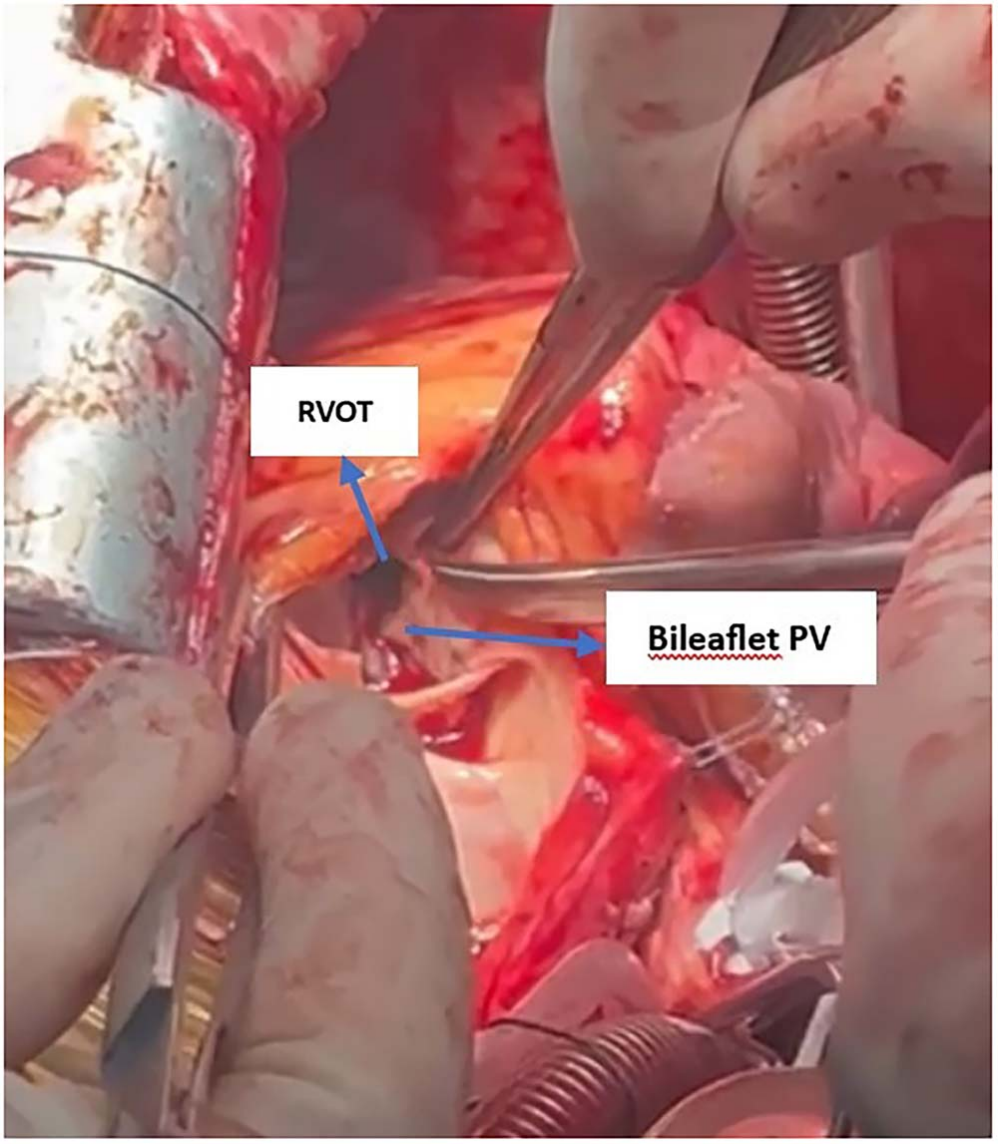
Intraoperative view showing the subvalvular RVOT with a bileaflet pulmonary valve. Severe obstruction at the RVOT is evident, indicated by narrowing below the valve leaflets.

Intraoperatively, the PV beneath the RVOT was small, dysplastic, and bileaflet, causing severe RVOT obstruction (Fig. 6). During RVOT resection, an unexpected artery was transected, which upon further inspection was identified as an anomalous LAD crossing anterior to the RVOT (Video 1). The proximal segment was ligated, and the distal segment was bypassed with a saphenous vein graft. RVOT reconstruction was performed with a Dacron patch, and the PV was replaced with a size 23 bioprosthetic valve. Additionally, the tricuspid valve was repaired. Temporary epicardial leads were placed on the RV, and the patient was transferred to the intensive care unit in stable condition.

Postoperatively, ascites decreased significantly. With normalization of liver enzymes and stable hemodynamics, the patient was discharged in NYHA functional class II. Follow-up echocardiography at 1 and 3 months demonstrated improvement in LV and RV function, adequate valvular performance, and resolution of RVOT obstruction. However, RV remained dilated and systolic function was impaired, along with RA enlargement. Pulmonary artery pressure decreased to a measurable level, although mild pulmonary hypertension persisted, necessitating continued monitoring (Table 1).

A timeline of key clinical interventions and events in provide (Table 2).

**Table 2:** Clinical timeline of key diagnostic and therapeutic events

Day	Event
28 April 2025	Hospital admission / initial evaluation
05 May 2025	Preoperative transesophageal echocardiography performed
16 May 2025	CT coronary angiography attempted (not feasible due to right ventricular outflow tract obstruction)
15 June 2025	Abdominal ultrasound / liver and inferior vena cava assessment
17 June 2025	Cardiac surgery (pericardiotomy, right ventricular outflow tract reconstruction, pulmonary valve replacement, tricuspid repair, left anterior descending artery bypass)
26 June 2025	Hospital discharge after postoperative recovery
25 July 2025	Postoperative echocardiography (follow-up)

## Discussion

This case represents a rare and complex combination of constrictive pericarditis, severe RVOT obstruction, TR, and anomalous LAD artery.

The RVOT obstruction in this patient was due to a congenital dysplastic pulmonary valve with associated muscular hypertrophy, leading to chronic right ventricular pressure overload, whereas constrictive pericarditis was an acquired condition confirmed histopathologically; no associated VSD or ASD was identified, and the presence of an anomalous LAD crossing the RVOT increased surgical complexity.

The coexistence of these lesions produced atypical clinical manifestations and complicated the preoperative differential diagnosis.

One important clinical feature in this patient was ascites and hepatic dysfunction secondary to congestive hepatopathy rather than primary liver disease, highlighting the importance of distinguishing primary hepatic pathology from secondary cardiac induced changes. In initial assessments, RV and RA enlargement with a small LV did not match the classic constrictive pericarditis pattern which typically features small atria and dilated ventricles and this unusual pattern complicated the diagnosis [6]. Moreover, although preoperative TEE demonstrated diastolic filling limitation, this finding was plausibly related to RV hypertrophy and pressure overload, and alone was insufficient to suggest constrictive pericarditis. This represents a key diagnostic pitfall, as reliance on TEE as a single modality may mask constrictive pericarditis preoperatively, making intraoperative discovery unexpected but plausible [1].

Pathological examination revealed non-granulomatous pericardial fibrosis with areas of granulation tissue consistent with constrictive pericarditis, distinct from granulomatous lesions such as tuberculosis, which have different diagnostic and therapeutic implications [2]. Negative adenosine deaminase and smear results also ruled out tuberculosis. Fibro-hyaline degenerative valvulopathy beneath the PV confirmed chronic pulmonary stenosis.

Preoperative coronary angiography is recommended for patients over 40 undergoing cardiac surgery [7]. In this case, the anomalous LAD was not identified preoperatively, leading to inadvertent transection during RVOT resection. Coronary CTA could have been a safer method for identifying this anomaly and preventing unexpected intraoperative injury [5]. Hypertrophic muscular bands in the RVOT and a small, dysplastic PV caused combined sub- and supravalvular obstruction, further complicating surgery. Although correction of these lesions improved symptoms and relieved right heart failure, complete RV functional recovery was not achieved (Table 1). Persistent reduction in tricuspid annular plane systolic excursion and ongoing systolic dysfunction indicate chronic and relatively irreversible RV remodeling. These findings are consistent with previous reports showing that in patients with chronic right heart failure, RV recovery may be incomplete even after correction of underlying lesions [8]. Long term monitoring of RV function following pericardiectomy and simultaneous correction of valvular and RVOT lesions is essential to accurately assess recovery or progression of RV dysfunction.

## Conclusion

Accurate preoperative assessment of coronary anatomy, particularly in patients with RVOT obstruction, is critical to prevent unexpected intraoperative injury.

Although small atria and dilated ventricles are classic features of constrictive pericarditis, these manifestations may not be evident in the presence of RVOT obstruction or PV stenosis, complicating preoperative diagnosis. Furthermore, diastolic filling limitation on preoperative TEE should not be attributed solely to right heart failure; constrictive pericarditis must remain a key differential diagnosis.

When conventional coronary angiography cannot delineate coronary anatomy accurately, coronary CTA should be used as an important diagnostic tool to identify potential anomalies and prevent unexpected intraoperative injury.

## Supplementary Material

Video_1_rjag132

Video_1_rjag132
