# Supplementary material for: Diagnostic pitfalls in constrictive pericarditis coexisting with right ventricular outflow tract obstruction and coronary artery anomaly
Source: J Surg Case Rep. 2026 Mar 22;2026(3):rjag132. doi: 10.1093/jscr/rjag132 (PMC13006047; doi:10.1093/jscr/rjag132)
Supplement: Video_1_rjag132 [file video_1_rjag132.docx]

**Video 1**. Intraoperative video showing RVOT resection. After opening the bileaflet PV, an unexpected LAD was transected. The artery was anomalously positioned, crossing anterior to the RVOT.
